# Machine learning based on body composition radiomics for predicting early recurrence in colorectal cancer: a multicenter study

**DOI:** 10.3389/fnut.2026.1843436

**Published:** 2026-06-04

**Authors:** Yongjie Zhou, Miaoping Zhou, Yongming Tan, Jinhong Zhao, Shengfa Zeng, Anni Yu, Sijia Dong, Lan Liu, Linhua Zhong

**Affiliations:** 1 Department of Radiology, Jiangxi Cancer Hospital & Institute, Jiangxi Clinical Research Center for Cancer, The Second Affiliated Hospital of Nanchang Medical College, Nanchang, China; 2 Department of Radiology, The Quzhou Affiliated Hospital of Wenzhou Medical University, Quzhou People’s Hospital, Zhejiang, China; 3 Department of Radiology, The First Affiliated Hospital, Jiangxi Medical College, Nanchang University, Nanchang, China; 4 Department of Radiology, The Second Affiliated Hospital, Jiangxi Medical College, Nanchang University, Nanchang, China; 5 Department of Radiology, Ningdu County Hospital of Traditional Chinese Medicine, Ganzhou, China

**Keywords:** body composition, colorectal cancer, early recurrence, machine learning, radiomics

## Abstract

**Background:**

Early recurrence (ER) in colorectal cancer (CRC) leads to dismal outcomes. Current pTNM staging fails to capture the host’s systemic pathophysiological status. We developed an interpretable machine learning (ML) model based on preoperative CT body composition radiomics to predict ER in CRC.

**Methods:**

This multicenter study enrolled 917 patients who underwent radical resection across three independent institutions, and the cohort was partitioned into a training set (*n* = 548) and two external test sets (*n* = 263 and *n* = 106). We extracted 1,896 radiomic features from four body composition compartments: skeletal muscle (SM), subcutaneous adipose tissue (SAT), intermuscular adipose tissue (IMAT), and visceral adipose tissue (VAT) at the L3 level on CT. Following feature selection using LASSO and Boruta algorithms, eight ML algorithms were evaluated. The optimal classifier was integrated with clinical risk factors. SHAP was utilized for model interpretability.

**Results:**

An 11-feature radiomics signature was identified. The Random Forest model demonstrated optimal generalization, yielding AUCs of 0.807, 0.776, and 0.750 in the training and two test sets, respectively. SHAP analysis revealed that IMAT (46.1%) and SM (42.9%) features were primary drivers, and increased SM textural uniformity may reflect adverse muscle quality and possible myosteatosis-related tissue alterations. The individualized radiomics risk score effectively stratified patients, demonstrating significantly divergent recurrence-free and overall survival across all cohorts (*p* < 0.05). Decision curve analysis confirmed superior net clinical benefit over pTNM staging.

**Conclusion:**

This interpretable ML model may improve ER risk stratification in CRC and provide a quantitative tool for individualized postoperative surveillance.

## 1 Introduction

Colorectal cancer (CRC) is a highly prevalent gastrointestinal malignancy, ranking third in incidence and second in mortality among all cancers worldwide (1). Despite advances in radical resection and adjuvant therapies, early recurrence (ER ≤ 24 months post-resection) remains a major barrier to long-term survival for patients with non-metastatic CRC (2). Current clinical risk stratification relies heavily on pathological TNM (pTNM) staging. However, this system primarily focuses on localized anatomical features, failing to capture the systemic host status that critically influences tumor evolution (3). Consequently, patients with identical pTNM stages often exhibit substantial heterogeneity in survival outcomes (4). This highlights an urgent clinical need for novel, quantitative biomarkers reflecting the host’s pathophysiological baseline to optimize early identification and personalized surveillance for high-risk populations.

Tumor recurrence is not merely a localized pathological event; rather, it depends heavily on the host’s metabolic and immunological microenvironment (5). Quantitative body composition analysis using routine abdominal computed tomography (CT) at the third lumbar vertebra (L3) provides a vital non-invasive window into this systemic pathophysiology (6). Emerging evidence indicates that adipose tissue functions as an active endocrine organ, promoting cancer progression through paracrine signaling and metabolic reprogramming of the tumor microenvironment (7). Concurrently, skeletal muscle depletion and fat infiltration are strongly linked to impaired immune surveillance (8). These mechanisms establish body composition alterations as biologically plausible and clinically modifiable risk factors. However, prior body composition evaluations have primarily relied on macroscopic metrics such as cross-sectional area or mean CT attenuation (9, 10). Although these conventional parameters effectively estimate tissue mass, they are fundamentally inadequate in characterizing intrinsic tissue quality (11, 12). They cannot adequately capture microscopic spatial heterogeneity, underlying adipose micro-inflammation, or early-stage myosteatosis, which represents an inherent methodological limitation.

Traditional tumor-centric prediction models rely on localized pathological features (13), neglecting the systemic metabolic and immunological status central to recurrence mechanisms. Moreover, tumor-derived radiomic features are highly susceptible to variations in scanner settings and segmentation protocols, compromising model reproducibility (14–16). Crucially, these localized features lack direct biological interpretability and offer no clear clinical intervention targets. In contrast, body composition radiomics may partly address these limitations (6). Recent studies in malignancies such as hepatocellular carcinoma and pancreatic cancer demonstrate that body composition radiomics captures microenvironmental remodeling information missed by conventional macroscopic metrics (12, 17, 18), significantly outperforming traditional clinical and body composition indices alone. However, in CRC, studies extracting high-dimensional radiomics features from multi-compartmental L3 body composition to systematically validate their independent predictive value for ER remain scarce. Additionally, the “black box” nature of machine learning (ML) algorithms limits the intuitive clinical interpretability of radiomics models, hindering their real-world clinical translation.

Consequently, this multicenter retrospective study extracted high-dimensional radiomics features from four core L3 body composition compartments and combined them with ML to construct a prediction model for ER of CRC. We integrated the SHapley Additive exPlanations (SHAP) framework to quantify the predictive contributions of these features. The primary objective of this study was to develop and validate an interpretable machine learning model based on preoperative CT body composition radiomics to predict ER in patients with CRC, thereby facilitating personalized risk stratification and informing postoperative surveillance.

## 2 Materials and methods

### 2.1 Study population and data collection

**Figure 1:**
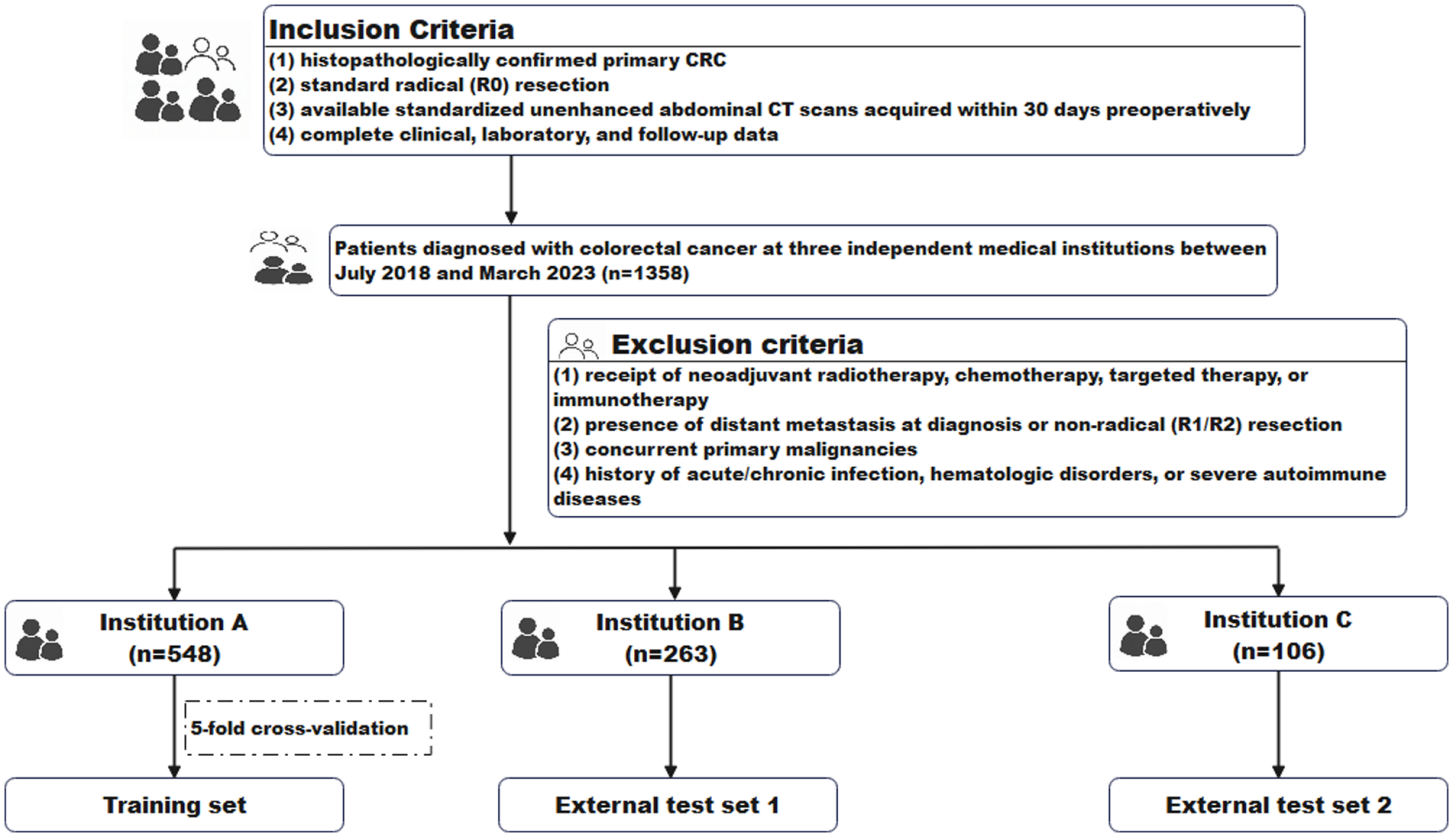
Flowchart of the study population selection process.

This retrospective study was approved by the Ethics Committee of Jiangxi Cancer Hospital (Approval No.: 2024ky071), and the requirement for informed consent was waived. The study consecutively enrolled patients with CRC who underwent surgical intervention at three independent medical institutions between July 2018 and March 2023. Based on the institutional source, the cohort was partitioned into a training set (*n* = 548 from Institution A) and two independent external test sets (*n* = 263 from Institution B as Test Set 1; *n* = 106 from Institution C as Test Set 2). The inclusion criteria were: (1) histopathologically confirmed primary CRC; (2) standard radical (R0) resection; (3) available standardized unenhanced abdominal CT scans acquired within 30 days preoperatively; and (4) complete clinical, imaging, laboratory, and follow-up data. Exclusion criteria included: (1) receipt of neoadjuvant radiotherapy, chemotherapy, targeted therapy, or immunotherapy; (2) presence of distant metastasis at diagnosis or non-radical (R1/R2) resection; (3) concurrent primary malignancies; or (4) history of acute/chronic infection, hematologic disorders, or severe autoimmune diseases. Because only patients with complete clinical, laboratory, imaging, and follow-up data were included in the final analysis, no missing-value imputation was performed. Figure 1 illustrates the patient selection flowchart.

Baseline demographics, clinicopathological characteristics, and laboratory findings were extracted from electronic medical records. Routine preoperative laboratory tests were performed within 1 week prior to surgery. Fasting peripheral venous blood samples were collected, and complete blood counts were analyzed using standard automated hematology analyzers. Serum carcinoembryonic antigen (CEA) levels were measured using standard chemiluminescence immunoassay methods. In addition to carcinoembryonic antigen and absolute peripheral blood counts, five systemic inflammatory-immunological indices were derived: neutrophil-to-lymphocyte ratio, platelet-to-lymphocyte ratio, monocyte-to-lymphocyte ratio, systemic immune-inflammation index, and systemic inflammation response index. Detailed calculation methods can be found in the Supplementary material. Pathological evaluation included primary tumor site, T stage, N stage, pTNM stage, histological type, differentiation grade, perineural invasion (PNI), and lymphovascular invasion (LVI). Postoperative surveillance was conducted every 3 months for the first 2 years and every 6 months thereafter, incorporating serial tumor markers, cross-sectional imaging (CT/MRI), and endoscopic examination when clinically indicated. The primary endpoint was ER, defined as radiologically or pathologically confirmed local recurrence or distant metastasis within 24 months post-resection. Tumor marker elevation alone was not considered recurrence unless supported by imaging, endoscopic, pathological, or clinical evidence. Secondary endpoints included recurrence-free survival (RFS), measured from surgery to recurrence, and overall survival (OS), defined as the interval from surgery to death from any cause. The final follow-up date was September 2025. Because complete follow-up data were required for inclusion, all patients included in the final analysis had available follow-up information for assessment of the primary endpoint.

### 2.2 Image preprocessing and radiomics feature extraction

**Figure 2:**
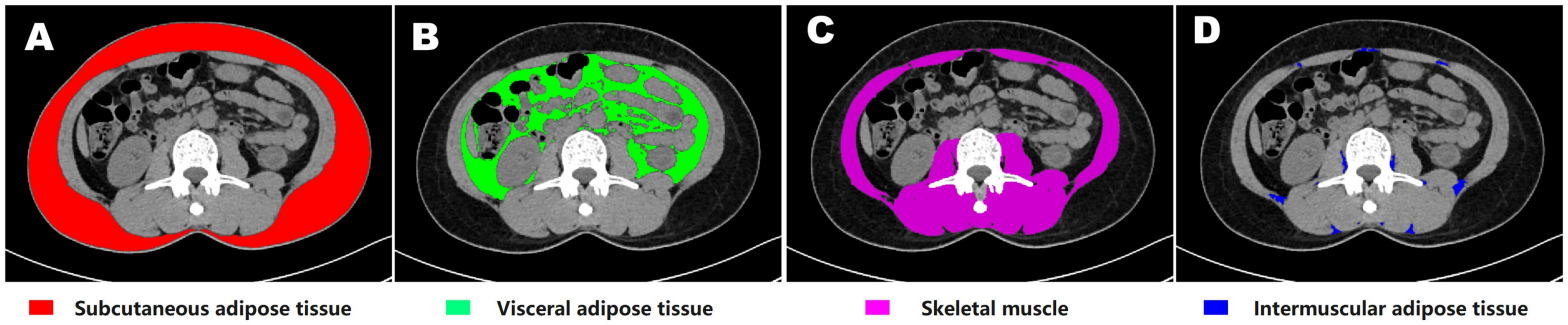
Representative CT images of body composition semi-automated segmentation at the third lumbar vertebra level. Four core body composition compartments were segmented based on specific Hounsfield unit thresholds: **(A)** Subcutaneous adipose tissue (red); **(B)** Visceral adipose tissue (green); **(C)** Skeletal muscle (purple); and **(D)** Intermuscular adipose tissue (blue).

Detailed information regarding the CT scanner models and image acquisition parameters across the three participating institutions is provided in Supplementary Table S1. Given that body composition at the L3 level highly correlates with total body tissue mass (19), a single-slice CT image at the L3 mid-vertebral level was utilized for quantitative analysis. Semi-automated segmentation of four core body composition compartments was performed using 3D Slicer (v5.4.0) based on validated Hounsfield unit (HU) thresholds (12) (Figure 2): skeletal muscle (SM, −29 to +150 HU), subcutaneous adipose tissue (SAT, −190 to −30 HU), intermuscular adipose tissue (IMAT, −190 to −30 HU), and visceral adipose tissue (VAT, −150 to −50 HU). All segmentations were performed by an abdominal radiologist with 11 years of experience (Radiologist A), who was blinded to clinical outcomes. To assess feature robustness, 30 patients were randomly selected for secondary independent segmentation by an abdominal radiologist with 13 years of experience (Radiologist B) after a four-week interval. Consistency was quantified using the intraclass correlation coefficient (ICC). Following B-spline interpolation resampling to a pixel size of 1*1 mm^2^ and grayscale discretization with a fixed bin width of 25, 2D radiomic features were extracted using the PyRadiomics library (20). The feature space encompassed 2D morphological (shape2D), first-order statistics, and high-order texture features derived from both original and wavelet-transformed images. Features from the four compartments were concatenated to construct a high-dimensional radiomic feature pool.

### 2.3 Feature selection

To prevent data leakage, feature engineering was conducted strictly within the training set. The feature matrix was initially standardized using Z-score normalization. Highly collinear features were subsequently removed based on Pearson correlation coefficients (|r| > 0.7). To identify the most discriminative and stable predictors, a tandem dual-reduction strategy was deployed: (1) Least Absolute Shrinkage and Selection Operator (LASSO) regression with 10-fold cross-validation was used to compress redundant variables based on the penalty parameter minimizing the area under the curve (*λ*.min); (2) the Boruta algorithm, a random forest-based wrapper method, was applied over 500 iterations to retain only features confirmed significant through comparison with shadow features. The intersection of variables selected by both algorithms was defined as the final core radiomics signature.

### 2.4 Model construction and interpretability

Eight ML algorithms, including k-nearest neighbors, support vector machine, decision tree, random forest (RF), adaptive boosting, GradientBoosting, extreme gradient boosting (XGBoost), and light gradient boosting machine, were employed to construct classification models for predicting ER. Hyperparameters were optimized via GridSearchCV combined with stratified 5-fold cross-validation. Sample imbalance was addressed by assigning class weights (class_weight = ‘balanced’). To ensure objective evaluation, the optimal cutoff value determined by the maximum Youden’s Index in the training set was fixed and applied to the two external test sets. Model performance was evaluated using receiver operating characteristic (ROC) curves and the area under the curve (AUC) with 95% confidence intervals (CI) calculated via 1,000-iteration bootstrap resampling. Additional metrics included accuracy, F1-score, sensitivity, specificity, and positive/negative predictive values (PPV/NPV).

For model interpretability, the SHAP framework was applied to the best-performing classifier (9). TreeExplainer was utilized to calculate sample-level SHAP values, quantifying the marginal contribution of each feature to the ER prediction. Global impact and feature importance were visualized using beeswarm and bar plots. Furthermore, risk stratification was performed by dividing the cohort into high- and low-risk groups based on the optimal cutoff. Survival differences were evaluated using Kaplan–Meier curves and Log-rank tests across clinical subgroups and according to adjuvant therapy status.

### 2.5 Combined model development and validation

To assess the incremental clinical value of radiomics features beyond traditional staging, four models were compared: (1) a baseline pTNM model; (2) a clinical model incorporating multiple clinicopathological risk factors; (3) an optimal radiomics-only machine learning model; and (4) an integrated model fusing independent clinical risk factors with machine learning-derived probabilities. In addition to AUC comparisons, calibration was assessed via calibration curves and Brier scores. Decision curve analysis (DCA) was utilized to quantify the clinical net benefit across a range of threshold probabilities.

### 2.6 Statistical analysis

Statistical analyses were performed using Python (version 3.11.14) with libraries including scikit-learn, lifelines, and SHAP. Sample size adequacy was established using the Events Per Variable (EPV) criterion rather than traditional hypothesis-driven calculations, which are less applicable to retrospective machine learning predictive models (21). To minimize the risk of overfitting, we adhered to the widely recommended EPV threshold of ≥ 10. In our training cohort, 117 ER events were observed. The final radiomics signature incorporated 11 core features, yielding an EPV of 10.6, which satisfied this statistical requirement. Furthermore, empirical validation across two independent external cohorts confirmed the model’s robustness and the adequacy of the sample size. Continuous variables were compared using the independent-sample *t*-test or the Mann–Whitney U test for two groups, and the Kruskal-Wallis test for three groups, depending on data distribution. Categorical variables were evaluated using the Chi-square test. Independent clinical predictors were identified via univariable followed by multivariable logistic regression. Survival rates between high- and low-risk groups were compared using Log-rank tests. All hypothesis tests were two-tailed, and *p* < 0.05 was considered statistically significant.

## 3 Results

### 3.1 Study population and clinical risk factors

A total of 917 patients who underwent radical resection for CRC were enrolled and partitioned into a training set (*n* = 548), external test set 1 (*n* = 263), and external test set 2 (*n* = 106). The ER rates were 21.35% (117/548), 20.91% (55/263), and 23.58% (25/106) across the three cohorts, respectively. As shown in Table 1, the median ages were 58 (IQR: 50–67), 57 (IQR: 50–66), and 60 (IQR: 51–68) years. Except for the monocyte count, no significant differences were observed in baseline clinicopathological characteristics among the three cohorts (all *p* value > 0.05), indicating a balanced distribution across institutions.

**Table 1:** Baseline characteristics of the population.

Characteristics	Training set (*n* = 548)	External test set 1 (*n* = 263)	External test set 2 (*n* = 106)	*p*-value*
Age (year)	58 [50, 67]	57 [50, 66]	60 [51, 68]	0.488
Gender				0.358
Male	221 (40.33)	120 (45.63)	45 (42.45)	
Female	327 (59.67)	143 (54.37)	61 (57.55)	
Smoking history				0.466
No	409 (74.64)	204 (77.57)	84 (79.25)	
Yes	139 (25.36)	59 (22.43)	22 (20.75)	
Alcohol history				0.156
No	467 (85.22)	210 (79.85)	88 (83.02)	
Yes	81 (14.78)	53 (20.15)	18 (16.98)	
BMI (kg/m^2^)				0.171
<18.5	112 (20.44)	71 (27.00)	28 (26.42)	
18.5 ~ 24.9	286 (52.19)	131 (49.81)	56 (52.83)	
≥25.0	150 (27.37)	61 (23.19)	22 (20.75)	
White blood cell	5.85 [4.76, 7.05]	5.88 [4.80, 7.22]	5.88 [5.01, 7.40]	0.587
Lymphocyte	1.48 [1.18, 1.87]	1.58 [1.24, 1.89]	1.44 [1.07, 1.81]	0.06
Monocyte	0.38 [0.30, 0.46]	0.41 [0.33, 0.53]	0.35 [0.29, 0.47]	<0.001
Platelet	243 [198, 306]	243 [200, 301]	250 [204, 309]	0.831
Neutrophil	3.73 [2.84, 4.62]	3.75 [2.87, 4.74]	3.94 [3.15, 5.11]	0.096
Tumor location				0.436
Colon	304 (55.47)	139 (52.85)	52 (49.06)	
Rectum	244 (44.53)	124 (47.15)	54 (50.94)	
CEA (ng/mL)				0.197
<5	395 (72.08)	177 (67.30)	69 (65.09)	
≥5	153 (27.92)	86 (32.70)	37 (34.91)	
T stage, *n* (%)				0.356
T1 – T2	75 (13.69)	44 (16.73)	19 (17.92)	
T3 – T4	473 (86.31)	219 (83.27)	87 (82.08)	
N stage				0.31
N0	281 (51.28)	128 (48.67)	46 (43.40)	
N1 – N2	267 (48.72)	135 (51.33)	60 (56.60)	
pTNM stage				0.217
I	58 (10.58)	35 (13.31)	14 (13.21)	
II	223 (40.69)	93 (35.36)	32 (30.19)	
III	267 (48.72)	135 (51.33)	60 (56.60)	
Pathological type				0.255
Adenocarcinoma	480 (87.59)	220 (83.65)	89 (83.96)	
Other histologic types	68 (12.41)	43 (16.35)	17 (16.04)	
Differentiation grade				0.236
Poorly	125 (22.81)	48 (18.25)	19 (17.92)	
Moderately-well	423 (77.19)	215 (81.75)	87 (82.08)	
Perineural invasion				0.831
No	441 (80.47)	207 (78.71)	84 (79.25)	
Yes	107 (19.53)	56 (21.29)	22 (20.75)	
Lymphovascular invasion				0.547
No	391 (71.35)	185 (70.34)	70 (66.04)	
Yes	157 (28.65)	78 (29.66)	36 (33.96)	
Postoperative adjuvant therapy				0.117
No	177 (32.30)	104 (39.54)	39 (36.79)	
Yes	371 (67.70)	159 (60.46)	67 (63.21)	
ER				0.846
No	431 (78.65)	208 (79.09)	81 (76.42)	
Yes	117 (21.35)	55 (20.91)	25 (23.58)	

Characteristics are presented as number (percentage), median (interquartile range). BMI, body mass index; CEA, carcinoembryonic antigen; pTNM, pathological TNM; ER, early recurrence. *p-values were calculated using the Kruskal-Wallis test for continuous variables (expressed as median [interquartile range]) and the Chi-square test for categorical variables (expressed as *n* (%)) to evaluate the differences among Institution A, Institution B, and Institution C.

Multivariable logistic regression analysis in the training set (Table 2) identified preoperative CEA (OR = 1.633, 95% CI: 1.023–2.588, *p* = 0.038), N stage (OR = 2.183, 95% CI: 1.369–3.523, *p* = 0.001), pathological type (OR = 2.387, 95% CI: 1.325–4.249, *p* = 0.003), and LVI (OR = 2.236, 95% CI: 1.364–3.662, *p* = 0.001) as independent risk factors for ER. Although advanced T stage (T3 – T4) was only borderline significant (OR = 2.558, 95% CI: 1.061–7.627, *p* = 0.057), it was retained in the clinical model due to its established prognostic relevance in CRC.

**Table 2:** Univariate and multivariate logistic regression analysis for predicting early recurrence of colorectal cancer.

Characteristics	Univariable		Multivariable	
	OR (95% CI)	*p*-value	OR (95% CI)	*p*-value
Age (years)	1.014 (0.995–1.033)	0.145		
Gender (male vs. female)	0.843 (0.558–1.274)	0.418		
Smoking history (yes vs. no)	1.078 (0.677–1.716)	0.751		
Alcohol history (yes vs. no)	0.892 (0.495–1.609)	0.704		
BMI (kg/m^2^)				
18.5 ~ 24.9	0.984 (0.573–1.692)	0.955		
≥25.0	1.222 (0.676–2.209)	0.507		
CEA (≥ 5 vs. < 5 ng/mL)	1.869 (1.214–2.878)	0.004	1.633 (1.023–2.588)	0.038
NLR	1.024 (0.993–1.055)	0.128		
PLR	1.001 (0.999–1.003)	0.184		
MLR	2.998 (1.092–8.230)	0.033	2.399 (0.620–9.037)	0.192
SII	1.000 (1.000–1.000)	0.097		
SIRI	1.076 (1.002–1.156)	0.043	1.022 (0.933–1.123)	0.630
Tumor location (rectum vs. colon)	1.240 (0.823–1.867)	0.304		
T stage (T3–4 vs. T1–2)	4.343 (1.711–11.028)	0.002	2.558 (1.061–7.627)	0.057
N stage (N1–2 vs. N0)	3.115 (2.008–4.834)	<0.001	2.183 (1.369–3.523)	0.001
Pathological type (other vs. adenocarcinoma)	2.452 (1.425–4.219)	0.001	2.387 (1.325–4.249)	0.003
Differentiation grade (moderately-well vs. poorly)	0.731 (0.458–1.166)	0.188		
Perineural invasion (yes vs. no)	2.524 (1.586–4.017)	<0.001	1.231 (0.705–2.120)	0.458
Lymphovascular invasion (yes vs. no)	3.000 (1.960–4.591)	<0.001	2.236 (1.364–3.662)	0.001

OR, odds ratio; CI, confidence interval; BMI, body mass index; CEA, carcinoembryonic antigen; NLR, neutrophil-to-lymphocyte ratio; PLR, platelet-to-lymphocyte ratio; MLR, monocyte-to-lymphocyte ratio; SII, systemic immune-inflammation index; SIRI, systemic inflammation response index.

### 3.2 Radiomics feature engineering and signature construction

A total of 1,896 high-dimensional 2D radiomics features were extracted from SM, SAT, IMAT, and VAT at the L3 level. After filtering for inter-observer reproducibility (ICC ≥ 0.75), Z-score normalization, and the removal of highly collinear features (|r| > 0.7), 156 candidates remained.

**Figure 3:**
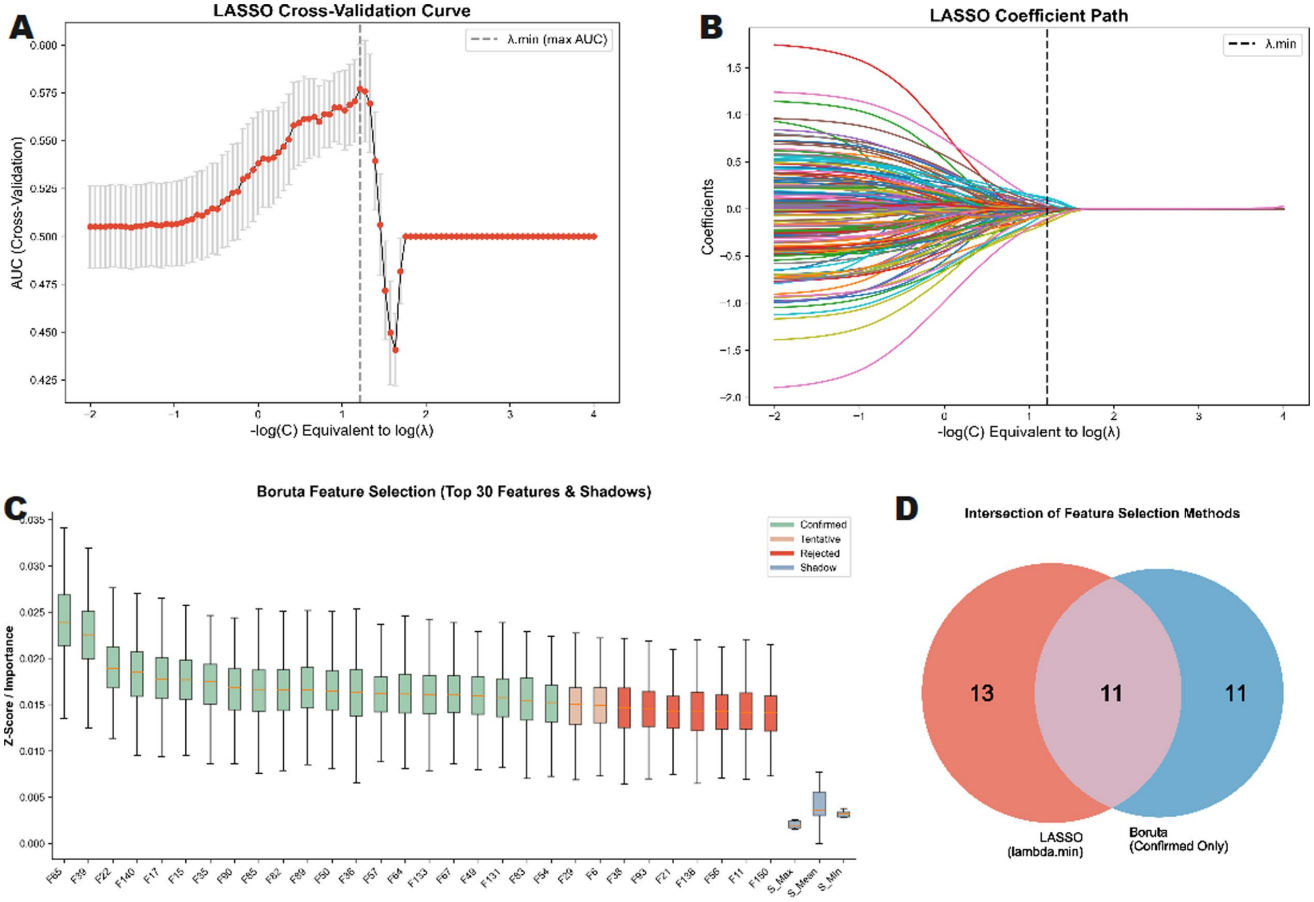
Radiomics feature selection using a tandem dual-reduction strategy. **(A)** Tuning parameter (*λ*) selection in the Least Absolute Shrinkage and Selection Operator (LASSO) model using 10-fold cross-validation via minimum criteria (λ.min). **(B)** LASSO coefficient path profiles of the radiomics features. **(C)** Feature selection using the Boruta algorithm, retaining features confirmed to be significantly more important than shadow features. **(D)** Venn diagram illustrating the intersection of features selected by both LASSO and Boruta algorithms, which yielded the final 11 body composition radiomics features.

A dual-reduction strategy was then applied to select robust predictors (Figure 3). First, LASSO regression with 10-fold cross-validation identified 24 features with non-zero coefficients at the optimal penalty parameter (*λ*.min) (Figures 3A,B). Second, the Boruta algorithm confirmed 22 significant features after 500 iterations (Figure 3C). The intersection of these methods yielded 11 core radiomics features, which were used to construct the final body composition radiomics signature (Figure 3D).

### 3.3 Performance comparison of machine learning classifiers

**Figure 4:**
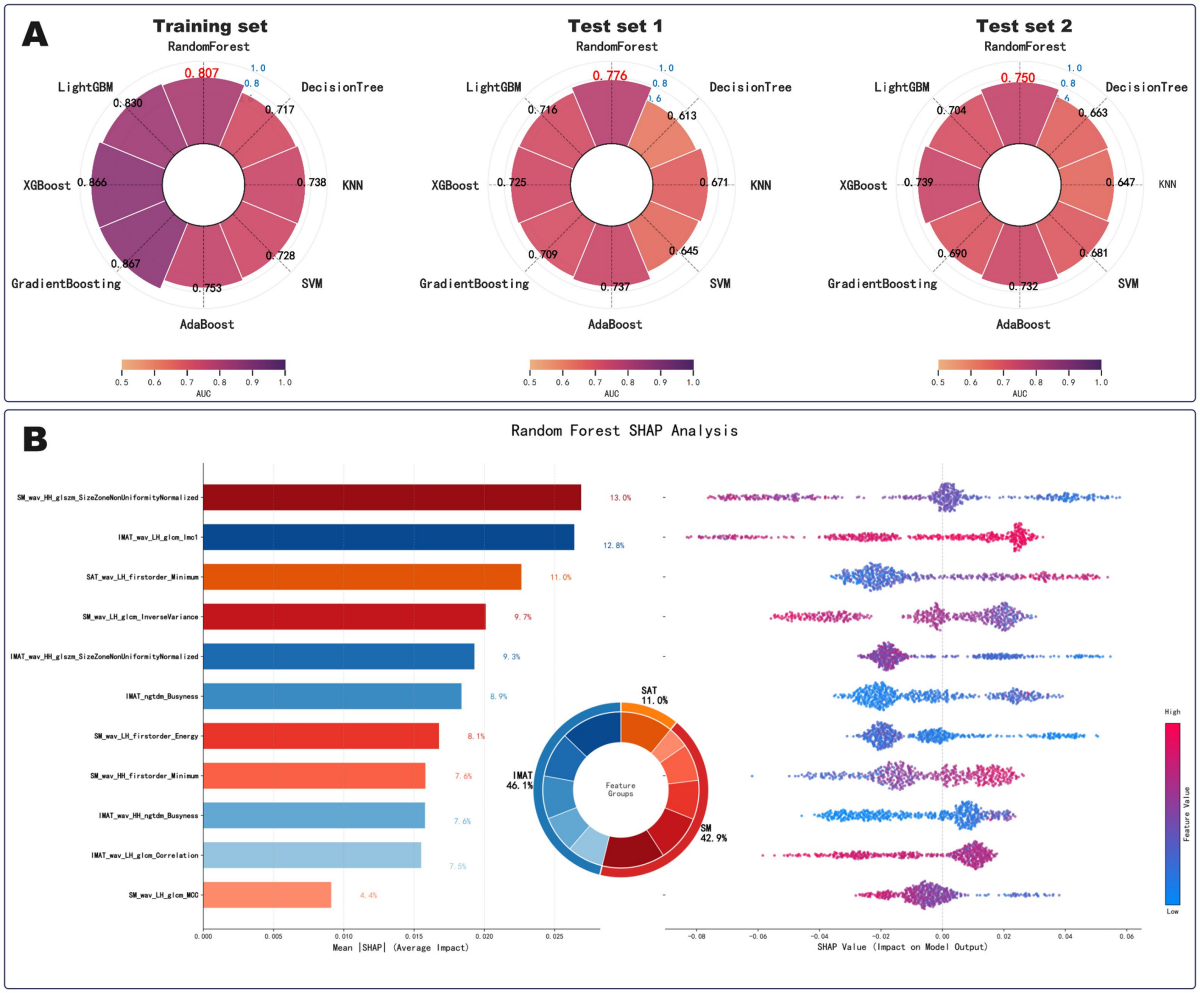
Performance evaluation of machine learning classifiers and model interpretability. **(A)** Radar charts displaying the area under the curve (AUC) of eight machine learning algorithms for predicting early recurrence in the training set, external test set 1, and external test set 2. The random forest model demonstrated the most consistent and optimal generalization. **(B)** SHapley additive explanations (SHAP) analysis of the optimal random forest model. The bar chart and inserted pie chart (left) illustrate the global importance and proportional contributions of features from different compartments (IMAT, SM, SAT). The beeswarm plot (right) depicts the distribution of sample-level SHAP values for each feature, demonstrating their directional impact on early recurrence risk (red indicates higher feature values, blue indicates lower values).

Eight ML algorithms were evaluated for their ability to predict ER (Table 3; Figure 4A). In the training set, GradientBoosting (AUC = 0.867) and XGBoost (AUC = 0.866) exhibited the highest discriminative performance, while the RF model achieved an AUC of 0.807 (95% CI: 0.766–0.846). During independent validation, the RF model demonstrated superior generalization and robustness. It achieved the highest predictive performance in external test set 1 (AUC = 0.776, 95% CI: 0.707–0.853; sensitivity = 0.836) and external test set 2 (AUC = 0.750, 95% CI: 0.633–0.852; sensitivity = 0.885). Given its consistent performance across both independent cohorts, RF was selected as the optimal classifier for the body composition-based ML model.

**Table 3:** Performance of different machine learning classifiers in predicting early recurrence of colorectal cancer.

Cohort	Model	AUC (95% CI)	ACC	Sensitivity	Specificity	PPV	NPV	F1 score
Train	GradientBoosting	0.867 (0.829–0.905)	0.808	0.769	0.819	0.536	0.929	0.632
	XGBoost	0.866 (0.830–0.902)	0.814	0.752	0.831	0.547	0.925	0.633
	LightGBM	0.830 (0.793–0.867)	0.774	0.735	0.784	0.480	0.916	0.581
	RandomForest	0.807 (0.766–0.846)	0.721	0.778	0.705	0.417	0.921	0.543
	AdaBoost	0.753 (0.707–0.795)	0.675	0.726	0.661	0.368	0.899	0.489
	KNN	0.738 (0.693–0.782)	0.606	0.803	0.552	0.328	0.912	0.465
	SVM	0.728 (0.680–0.776)	0.692	0.667	0.698	0.375	0.885	0.480
	DecisionTree	0.717 (0.671–0.760)	0.566	0.855	0.487	0.312	0.925	0.457
Test 1	RandomForest	0.776 (0.707–0.853)	0.593	0.836	0.529	0.319	0.924	0.462
	AdaBoost	0.737 (0.658–0.817)	0.597	0.745	0.558	0.308	0.892	0.436
	XGBoost	0.725 (0.647–0.805)	0.681	0.691	0.678	0.362	0.892	0.475
	LightGBM	0.716 (0.638–0.795)	0.635	0.636	0.635	0.315	0.868	0.422
	GradientBoosting	0.709 (0.633–0.787)	0.570	0.745	0.524	0.293	0.886	0.421
	KNN	0.671 (0.592–0.753)	0.490	0.782	0.413	0.261	0.878	0.391
	SVM	0.645 (0.560–0.732)	0.624	0.582	0.635	0.296	0.852	0.393
	DecisionTree	0.613 (0.541–0.685)	0.468	0.873	0.361	0.265	0.915	0.407
Test 2	RandomForest	0.750 (0.633–0.852)	0.481	0.885	0.350	0.307	0.903	0.455
	XGBoost	0.739 (0.621–0.842)	0.519	0.885	0.400	0.324	0.914	0.474
	AdaBoost	0.732 (0.600–0.839)	0.462	0.846	0.338	0.293	0.871	0.436
	LightGBM	0.704 (0.586–0.810)	0.491	0.885	0.362	0.311	0.906	0.460
	GradientBoosting	0.690 (0.559–0.806)	0.443	0.846	0.312	0.286	0.862	0.427
	SVM	0.681 (0.552–0.797)	0.528	0.731	0.463	0.306	0.841	0.432
	DecisionTree	0.663 (0.550–0.777)	0.396	0.885	0.237	0.274	0.864	0.418
	KNN	0.647 (0.540–0.755)	0.462	0.962	0.300	0.309	0.960	0.467

AUC, area under the curve; CI, confidence interval; ACC, accuracy; PPV, positive predictive value; NPV, negative predictive value; KNN, k-nearest neighbors; SVM, support vector machine; XGBoost, extreme gradient boosting; LightGBM, light gradient boosting machine; AdaBoost, adaptive boosting.

### 3.4 Interpretability and risk stratification

The SHAP framework was utilized to quantify feature contributions and enhance model transparency (Figure 4B). The analysis revealed that the predictive power was primarily driven by features from the IMAT (46.1%) and SM (42.9%) compartments. Specifically, the skeletal muscle feature SM_wav_HH_glszm_SizeZoneNonUniformityNormalized provided the highest marginal contribution; lower values of this feature (blue points) were associated with a positive SHAP value, indicating an increased risk of ER. Additionally, high expression of the IMAT feature IMAT_wav_LH_glcm_Imc1 correlated with a higher recurrence probability, whereas higher values of the SAT feature SAT_wav_LH_firstorder_Minimum (red points) were linked to increased risk.

**Figure 5:**
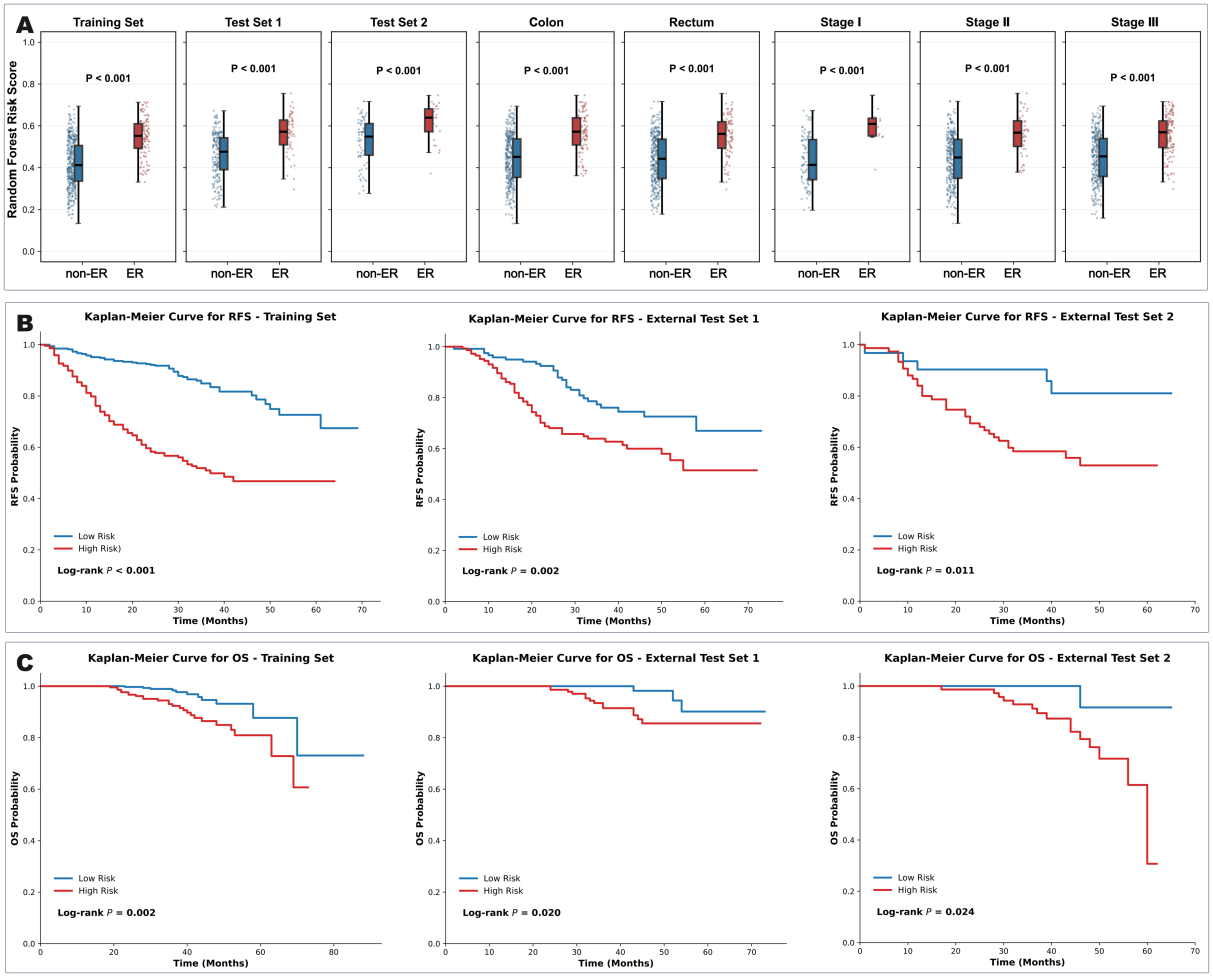
Risk stratification and survival analysis based on the optimal radiomics machine learning model. **(A)** Boxplots comparing the distribution of Random Forest (RF) risk scores between patients with and without early recurrence (ER) across different datasets, tumor locations, and pTNM stages. Kaplan–Meier curves for recurrence-free survival (RFS) **(B)** and overall survival (OS) **(C)**. Patients were stratified into high-risk and low-risk groups based on the RF model score, revealing significant survival differences across the training and two external validation sets (all Log-rank *p* < 0.05).

Individualized radiomics risk scores were derived based on the RF model’s probabilities. As illustrated in Figure 5A, patients with ER had significantly higher risk scores than those without ER across all clinical subgroups, including datasets, tumor locations, and pTNM stages (all *p* < 0.001). Kaplan–Meier survival analysis and Log-rank tests (Figures 5B,C) confirmed that the high-risk group had significantly shorter RFS and OS than the low-risk group in all three cohorts (all *p* < 0.05). Subgroup analyses (Supplementary Figure S1) further demonstrated that the risk stratification remained robust regardless of whether patients received adjuvant therapy. Univariable and multivariable Cox regression analyses further confirmed the independent prognostic value of the radiomics risk stratification for RFS. After adjustment for clinicopathological factors, the high-risk group remained independently associated with shorter RFS (HR = 3.626, 95% CI: 2.552–5.152, *p* < 0.001; Supplementary Table S2).

### 3.5 Incremental value and clinical utility of the integrated model

**Figure 6:**
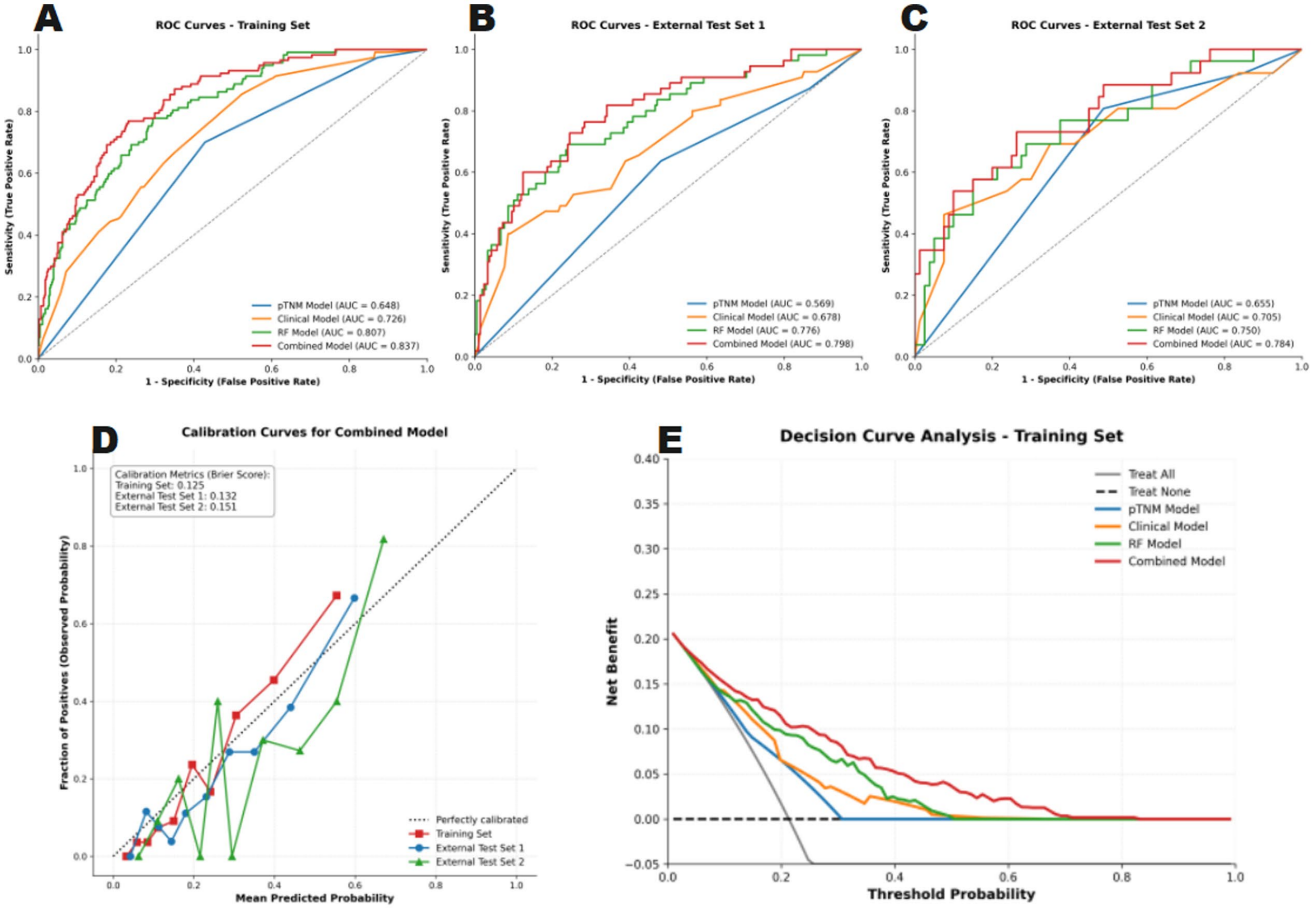
Incremental value and clinical utility of the combined model. **(A–C)** Receiver operating characteristic (ROC) curves comparing the predictive performance of the pTNM model, clinical model, radiomics (RF) model, and combined model in the training set **(A)**, external test set 1 **(B)**, and external test set 2 **(C)**. **(D)** Calibration curves of the combined model across all three cohorts, demonstrating strong agreement between the predicted probabilities and the observed early recurrence frequencies. **(E)** Decision curve analysis (DCA) in the training set evaluating the clinical net benefit across a wide range of threshold probabilities, highlighting the superiority of the combined model over traditional pTNM staging and clinical models.

To evaluate the incremental value of body composition radiomics, four models were compared: pTNM staging, the clinical model, the radiomics-only ML model, and the combined model. As shown in the ROC curves (Figures 6A–C), the combined model achieved the highest predictive performance across all cohorts, with AUCs of 0.837, 0.798, and 0.784 in the training, external test 1, and external test 2 sets, respectively. The combined model outperformed both the pTNM and clinical models, underscoring the added prognostic value of body composition radiomics. Calibration curves (Figure 6D) demonstrated strong agreement between the predicted probabilities of the integrated model and the observed ER frequencies. Furthermore, DCA (Figure 6E) indicated that the combined model provided a higher net clinical benefit than either the pTNM staging or the clinical model across a wide range of threshold probabilities, highlighting its potential utility as a clinical decision-support tool.

## 4 Discussion

Predicting ER after radical resection for non-metastatic CRC remains challenging. In this multicenter study, we developed an integrated ML model combining clinical risk factors with L3 body composition radiomics. The RF algorithm demonstrated optimal generalization across two independent external cohorts (AUCs: 0.750 and 0.776). SHAP analysis identified features from IMAT and SM as the primary predictive drivers. These findings highlight routine preoperative CT as a quantitative platform to decode the host’s systemic pathophysiological status, providing substantial incremental prognostic value over conventional pTNM staging. Importantly, because abdominal CT is already a mandatory component of the standard preoperative workup for CRC, our model leverages pre-existing data without imposing any additional radiation exposure or financial burden. While the pTNM system is purely tumor-centric, our model complements it by decoding the critical host microenvironment, thereby explaining the heterogeneous recurrence outcomes among patients with identical pTNM stages.

The inherent limitations of the traditional pTNM staging system have driven the extensive exploration of alternative prognostic methodologies across various malignancies. For instance, the Immunoscore has been internationally validated as a robust prognosticator in colon cancer (4), demonstrating that quantifying *in situ* immune infiltrates can often outstrip the prognostic accuracy of the TNM system. Additionally, liquid biopsies utilizing circulating tumor DNA (ctDNA) have recently emerged as a paradigm-shifting molecular tool to detect minimal residual disease and redefine recurrence risk independently of standard staging (22, 23). Similarly, deep learning-based classifiers incorporating pathological markers are increasingly utilized to enhance post-surgical risk stratification (13). While these emerging alternative methods offer profound prognostic value, they are predominantly tumor-centric and rely heavily on the availability of postoperative pathological specimens or post-surgical blood draws. In contrast, our body composition radiomics model leverages routine, non-invasive preoperative CT imaging. By decoding the host’s systemic pathophysiological baseline rather than localized tumor characteristics, our approach provides a complementary, preemptive tool that can guide clinical decision-making and metabolic prehabilitation even before surgical intervention.

Adverse body composition phenotypes, such as sarcopenia and visceral adiposity, are established negative prognosticators in surgical oncology (24, 25). However, previous risk stratification models have predominantly relied on macroscopic metrics such as cross-sectional area and mean CT attenuation. While these parameters are clinically interpretable, they offer limited insight into intratissue heterogeneity and tissue quality, resulting in unsatisfactory predictive performance, with reported AUC values generally ranging from 0.60 to 0.68 (26, 27). Furthermore, traditional tumor-centric radiomics are frequently constrained by tumor boundary ambiguity and inter-scanner variability (14, 28, 29). In contrast, L3 skeletal muscle and adipose compartments offer anatomically stable and highly reproducible targets (6). In the present study, we used a standardized and widely adopted radiomics workflow, including LASSO for redundancy reduction, Boruta for robust wrapper-based feature selection, established machine-learning classifiers, and SHAP for model interpretation. Although this pipeline does not represent a new algorithmic development, its use improves methodological transparency, reproducibility, and comparability across institutions. The principal contribution of our study lies in applying this reproducible framework to multicompartmental L3 body composition radiomics and validating its incremental prognostic value for ER prediction in two independent external cohorts. By leveraging high-throughput radiomics, our study quantified intrinsic textural alterations within the host-related body composition compartments that may precede macroscopic volume loss, thereby addressing limitations of conventional morphological measurements.

The inherent “black-box” opacity of advanced ML models often restricts their clinical translation. Utilizing the SHAP framework helped relate model predictions to radiomic features with plausible biological relevance. In our model, IMAT and SM features accounted for nearly 90% of the predictive contribution. The top-ranking feature, SM_wav_HH_glszm_SizeZoneNonUniformityNormalized, characterizes spatial textural heterogeneity. As demonstrated by our SHAP analysis, lower values of this feature, which indicate increased textural uniformity within the muscle compartment, are associated with higher ER risk, potentially reflecting adverse muscle quality and possible myosteatosis-related tissue alterations. Pathophysiologically, myosteatosis involves ectopic lipid infiltration that disrupts the musculoskeletal secretory profile. This process is known to downregulate protective myokines (e.g., IL-15) essential for CD8 + T cell activation, while upregulating pro-inflammatory cytokines such as IL-6 and TNF-*α* (30, 31). Consequently, this chronic systemic inflammation fosters an immunosuppressive niche highly permissive to micrometastasis.

Similarly, the high predictive weight of the IMAT feature (IMAT_wav_LH_glcm_Imc1) highlights the detrimental role of ectopic fat deposition. IMAT functions as a metabolically active endocrine organ; its aberrant accumulation is strongly associated with insulin resistance and adipokine dysregulation (e.g., altered leptin/adiponectin ratios) (32). This metabolic shift acts as a key mediator in the adipose-muscle-tumor crosstalk, accelerating cancer progression (33). Furthermore, higher values of the SAT feature (SAT_wav_LH_firstorder_Minimum) were also positively correlated with an increased risk of ER. While subcutaneous fat is often considered less metabolically harmful than visceral fat, qualitative deterioration within the SAT compartment, such as adipocyte hypertrophy, tissue hypoxia, and localized micro-inflammation, can actively promote systemic metabolic dysfunction (34). Higher values of this radiomic feature may serve as a macroscopic surrogate for such adverse microstructural alterations. Once the expansion limit of SAT is reached and it becomes dysfunctional, its capacity to act as a protective metabolic sink is compromised (35). This dysfunction not only exacerbates systemic pro-inflammatory signaling but also promotes lipotoxic spillover into ectopic depots such as IMAT, thereby synergistically fostering an immunosuppressive and recurrence-permissive microenvironment (7).

Recognizing tissue functional quality as a determinant of ER risk carries significant clinical implications. Within the context of our validated cohorts, DCA demonstrated that our integrated model provided a higher predictive clinical net benefit across a wide range of threshold probabilities compared to standard pTNM staging. The individualized radiomics risk score successfully stratified patients with distinctly divergent recurrence-free and overall survival rates across all clinicopathological subgroups. Patients identified as high-risk harbor a permissive host microenvironment at the time of surgery, suggesting a potential need for more individualized postoperative surveillance. Clinically, identifying these high-risk patients may support consideration of closer postoperative surveillance and individualized adjuvant-treatment assessment. Moreover, it highlights the critical need for personalized prehabilitation strategies. Current evidence suggests that targeted perioperative interventions, encompassing resistance training and tailored nutritional support, can effectively mitigate myosteatosis and modulate systemic inflammatory responses (36, 37). Integrating body composition radiomics into the clinical workflow could thus serve as a triage tool to identify optimal candidates for these metabolic-targeted interventions.

Several limitations of the present study merit consideration. First, the retrospective design may have introduced selection bias and residual confounding. In particular, detailed information on prior or concomitant nutritional interventions, dietary modification, prehabilitation, exercise programs, or perioperative nutritional support was not consistently available across centers. These unmeasured factors may influence skeletal muscle quality, adipose tissue distribution, systemic inflammatory status, and body composition radiomic features. Therefore, prospective studies with standardized collection of nutritional and intervention-related data are needed to validate our findings. Second, the semi-automated segmentation of body composition compartments remains a time-consuming barrier to routine clinical integration. Future implementation of deep learning-based, fully automated segmentation networks (38) will be essential to streamline this process. Third, our analysis relied on a single, static preoperative CT scan. Longitudinal evaluations of body composition trajectories during the perioperative and adjuvant treatment phases could yield more dynamic prognostic insights. Finally, given that the study population was predominantly of Asian descent, cross-racial external validation in diverse global cohorts is necessary to ensure the universal applicability of the identified radiomics signature.

In conclusion, body composition radiomics effectively refines ER prediction in CRC by quantifying systemic pathophysiology rather than relying solely on localized tumor characteristics. The developed interpretable ML model suggests that muscle- and adipose-related radiomic heterogeneity may provide prognostic information beyond conventional staging. By offering substantial incremental prognostic value over traditional staging, this quantitative tool holds considerable promise for optimizing individualized postoperative surveillance and directing metabolic-targeted interventions in CRC management.

## Data Availability

The original contributions presented in the study are included in the article/Supplementary material, further inquiries can be directed to the corresponding authors.
