# Supplementary material for: Machine learning based on body composition radiomics for predicting early recurrence in colorectal cancer: a multicenter study
Source: Front Nutr. 2026 Jun 4;13:1843436. doi: 10.3389/fnut.2026.1843436 (PMC13275260; doi:10.3389/fnut.2026.1843436)
Supplement: Supplementary file 1 [file Data_Sheet_1.docx]

**Supplementary Material**

1. ***CT scanning protocol***

Given the study's temporal span, images were acquired on multiple scanners. All patients with colorectal cancer (CRC) underwent non-contrast CT examinations within one month before surgery, with the scanning range covering the region from the diaphragm to the pelvic floor. Detailed CT scanning protocols of each institution are summarized in Table S1.

Table S1. CT scanning protocol for patients with CRC.

| Manufacture | SIEMENS | SIEMENS | SIEMENS | Philips | Philips | Toshiba |
| --- | --- | --- | --- | --- | --- | --- |
| CT scanner | CT256 | CT128 | CT128 | CT 256 | CT256 | CT320 |
| Scanner mode | SOMATOM Drive | SOMATOM Definition AS | SOMATOM Definition Edge | Brilliance iCT | IQon Spectral CT | Aquilion ONE |
| Tube voltage (kV) | 120 | 120 | 120 | 120 | 120 | 120 |
| Tube current (mA) | 250 | 250 | 200 | 300-350 | 300 | 200 |
| Rotation time (s) | 0.5 | 0.5 | 0.5 | 0.5 | 0.4 | 0.5 |
| Collimation (mm) | 64*0.6 | 64*0.6 | 64*0.6 | 128*0.625 | 64*0.625 | 160*0.625 |
| Slice thickness (mm) | 1.25 | 2 | 1 | 2 | 1 | 3 |
| Matrix | 512 × 512 | 512 × 512 | 512 × 512 | 512 × 512 | 512 × 512 | 512 × 512 |
| Field of view (mm) | 350 | 350 | 350 | 350 | 350 | 350 |
| Kernel | Standard | Standard | B30f | Standard | Standard | Fc10 |

CT, computed tomography; CRC, colorectal cancer.

1. ***Calculation of combined variables related to clinical laboratory parameters***

Composite indices were calculated as follows[1]: neutrophil-to-lymphocyte ratio = neutrophil count / lymphocyte count; platelet-to-lymphocyte ratio = platelet count / lymphocyte count; monocyte-to-lymphocyte ratio =monocyte count / lymphocyte count; systemic immune-inflammation index = platelet count * neutrophil count / lymphocyte count; systemic inflammation response index = neutrophil count * monocyte count / lymphocyte count;

1. ***Subgroup analysis based on postoperative adjuvant therapy***


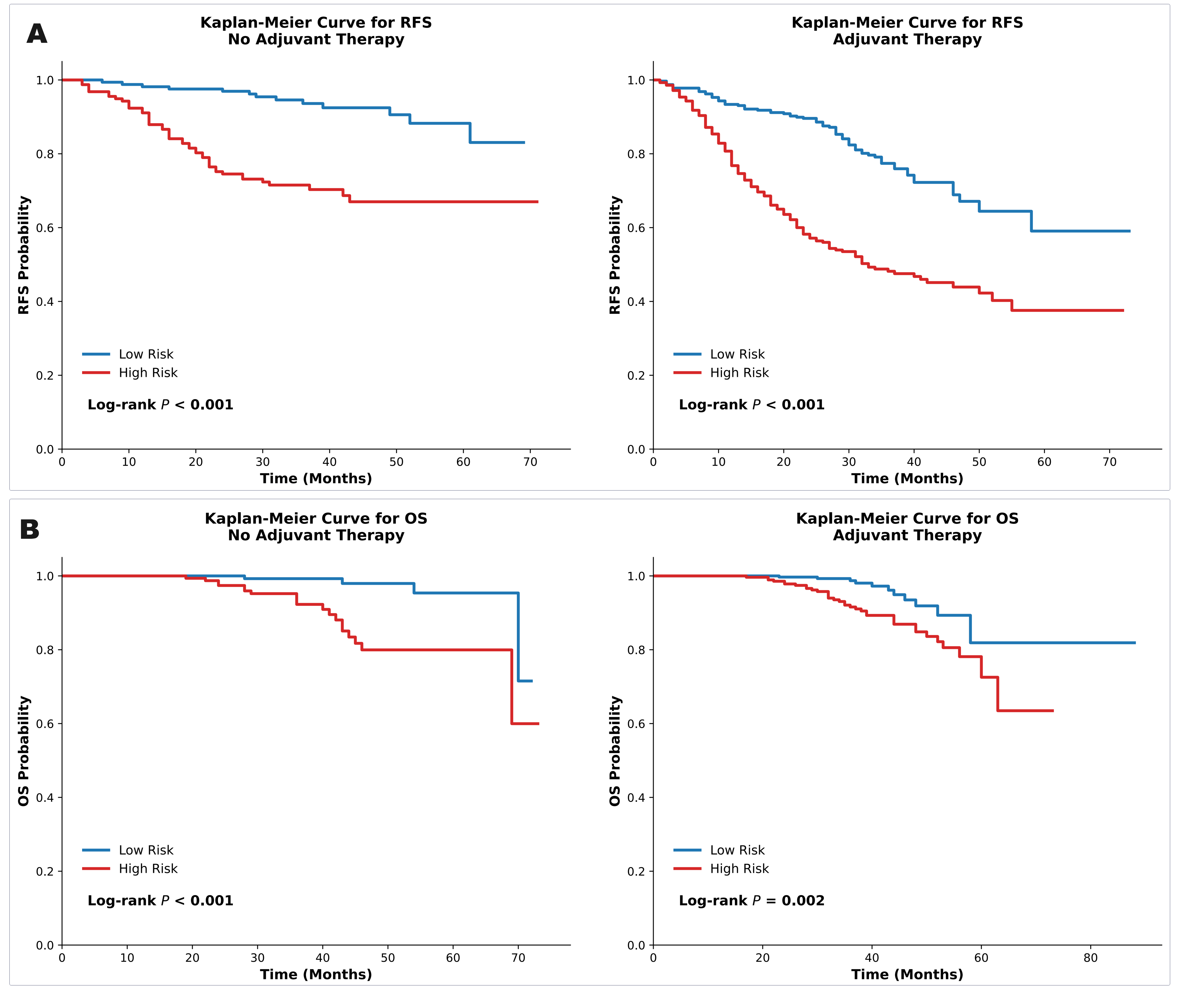


**Figure S1. Subgroup survival analysis based on adjuvant therapy status.** Kaplan-Meier curves for Recurrence-Free Survival (RFS) (A) and Overall Survival (OS) (B) stratified by the optimal radiomics machine learning model's risk score (High Risk vs. Low Risk) in patient subgroups who did not receive adjuvant therapy (left panels) and those who received adjuvant therapy (right panels). The radiomics-based risk stratification remained robust and statistically significant regardless of adjuvant therapy status (all Log-rank P < 0.05).

1. ***Univariate and multivariate Cox regression analysis for recurrence-free survival.***

| Table S2. Univariate and multivariate Cox regression analysis for recurrence-free survival. | | | | |
| --- | --- | --- | --- | --- |
| **Characteristics** | **Univariable** | | **Multivariable** | |
|  | **HR (95% CI)** | ***p-value*** | **HR (95% CI)** | ***p-value*** |
| Age (years) | 1.016 (1.001-1.030) | 0.034 |  |  |
| Gender (male vs female) | 1.024 (0.745-1.408) | 0.883 |  |  |
| Smoking history (yes vs no) | 1.236 (0.877-1.742) | 0.227 |  |  |
| Alcohol history (yes vs no) | 1.078 (0.703-1.652) | 0.732 |  |  |
| BMI (kg/m2) |  |  |  |  |
| 18.5～24.9 | 0.916 (0.670-1.251) | 0.580 |  |  |
| ≥ 25.0 | 1.214 (0.865-1.705) | 0.262 |  |  |
| CEA (≥ 5 vs < 5 ng/mL) | 1.641 (1.185-2.273) | 0.003 | 1.347 (0.966-1.879) | 0.079 |
| NLR | 1.016 (0.999-1.034) | 0.066 |  |  |
| PLR | 1.001 (1.000-1.003) | 0.066 |  |  |
| MLR | 2.207 (1.156-4.214) | 0.016 | 2.727 (1.145-6.499) | 0.024 |
| SII | 1.000 (1.000-1.000) | 0.087 |  |  |
| SIRI | 1.055 (1.013-1.098) | 0.009 | 0.990 (0.936-1.047) | 0.721 |
| Tumor location (rectum vs colon) | 1.089 (0.797-1.489) | 0.591 |  |  |
| T stage (T3-4 vs T1-2) | 2.619 (1.380-4.969) | 0.003 | 1.670 (0.869-3.210) | 0.124 |
| N stage (N1-2 vs N0) | 2.521 (1.803-3.526) | <0.001 | 1.697 (1.191-2.418) | 0.003 |
| Pathological type (other vs adenocarcinoma) | 1.945 (1.312-2.881) | <0.001 | 1.876 (1.260-2.794) | 0.002 |
| Differentiation grade (moderately-well vs poorly) | 0.649 (0.462-0.911) | 0.013 | 0.999 (0.688-1.449) | 0.994 |
| Perineural invasion (yes vs no) | 2.344 (1.681-3.268) | <0.001 | 1.278 (0.881-1.854) | 0.196 |
| Lymphovascular invasion (yes vs no) | 2.763 (2.020-3.781) | <0.001 | 2.098 (1.478-2.979) | <0.001 |
| Radiomics risk score (high-risk vs low-risk group) | 3.974 (2.850-5.540) | <0.001 | 3.703 (2.642-5.188) | <0.001 |
| HR, hazard ratio; CI, confidence interval; BMI, body mass index; CEA, carcinoembryonic antigen; NLR, neutrophil-to-lymphocyte ratio; PLR, platelet-to-lymphocyte ratio; MLR, monocyte-to-lymphocyte ratio; SII, systemic immune-inflammation index; SIRI, systemic inflammation response index. Multivariable Cox regression included the radiomics risk group and variables retained in the multivariable clinical model. | | | | |

**References:**

1 Zhu J, Wang D, Liu C et al (2023) Development and validation of a new prognostic immune-inflammatory-nutritional score for predicting outcomes after curative resection for intrahepatic cholangiocarcinoma: A multicenter study. Frontiers in Immunology 14:1165510
